# Personally perceived publication pressure: revising the Publication Pressure Questionnaire (PPQ) by using work stress models

**DOI:** 10.1186/s41073-019-0066-6

**Published:** 2019-04-09

**Authors:** Tamarinde L. Haven, Marije Esther Evalien de Goede, Joeri K. Tijdink, Frans Jeroen Oort

**Affiliations:** 1 Department of Philosophy, Vrije Universiteit Amsterdam, De Boelelaan 1105, 1081 HV Amsterdam, The Netherlands; 2 Centre for Applied Research on Economics & Management - CAREM, Postbus 814, 1000 AV Amsterdam / Wibautstraat 3b, 1091 GH Amsterdam, The Netherlands; 3 Department of Medical Ethics, University Medical Center Amsterdam, location VUmc, De Boelelaan, 1007 HV Amsterdam, The Netherlands; 4 Research Institute of Child Development and Education, University of Amsterdam, Nieuwe Achtergracht 127, 1018 WS Amsterdam, The Netherlands

**Keywords:** Publication pressure, Burnout, Research integrity, Validity, Reliability

## Abstract

**Background:**

The emphasis on impact factors and the quantity of publications intensifies competition between researchers. This competition was traditionally considered an incentive to produce high-quality work, but there are unwanted side-effects of this competition like publication pressure. To measure the effect of publication pressure on researchers, the Publication Pressure Questionnaire (PPQ) was developed. Upon using the PPQ, some issues came to light that motivated a revision.

**Method:**

We constructed two new subscales based on work stress models using the facet method. We administered the revised PPQ (PPQr) to a convenience sample together with the Maslach Burnout Inventory (MBI) and the Work Design Questionnaire (WDQ). To assess which items best measured publication pressure, we carried out a principal component analysis (PCA). Reliability was sufficient when Cronbach’s alpha > 0.7. Finally, we administered the PPQr in a larger, independent sample of researchers to check the reliability of the revised version.

**Results:**

Three components were identified as ‘stress’, ‘attitude’, and ‘resources’. We selected 3 × 6 = 18 items with high loadings in the three-component solution. Based on the convenience sample, Cronbach’s alphas were 0.83 for stress, 0.80 for attitude, and 0.76 for resources. We checked the validity of the PPQr by inspecting the correlations with the MBI and the WDQ. Stress correlated 0.62 with MBI’s emotional exhaustion. Resources correlated 0.50 with relevant WDQ subscales. To assess the internal structure of the PPQr in the independent reliability sample, we conducted the principal component analysis. The three-component solution explains 50% of the variance. Cronbach’s alphas were 0.80, 0.78, and 0.75 for stress, attitude, and resources, respectively.

**Conclusion:**

We conclude that the PPQr is a valid and reliable instrument to measure publication pressure in academic researchers from all disciplinary fields. The PPQr strongly relates to burnout and could also be beneficial for policy makers and research institutions to assess the degree of publication pressure in their institute.

**Electronic supplementary material:**

The online version of this article (10.1186/s41073-019-0066-6) contains supplementary material, which is available to authorized users.

## Background

Scientific output (publications) is the standard performance criterion for individual researchers and research institutions at large [1]. The rising prestige of impact factors and emphasis on quantity (of publications) intensifies competition between researchers [2]. This competition was traditionally considered an incentive to produce high-quality work, but in practice, there are also unwanted effects of this hyper-competitive and demanding publication climate in which you are mainly evaluated by the number of publications. This can result in (perceived) publication pressure [3]. Publication pressure is studied for its effects on research integrity as the pressure to publish may persuade researchers to cut corners [4, 5]. Publication pressure has also been linked to burnout in senior researchers as well as drop-out of academia among junior researchers [6, 7].

To measure these effects on research and researchers, the Publication Pressure Questionnaire (henceforth PPQ) was developed [8]. The PPQ aimed to assess publication pressure as perceived by biomedical researchers and has been used to measure publication pressure in both The Netherlands and Belgium. Publication pressure was related to burnout and associated with scientific misconduct [9, 10].

The PPQ was the first instrument to measure publication pressure in biomedical researchers. Upon using the PPQ in various studies, a few methodological limitations came to light: (1) the relation between the PPQ and burnout is moderate, (2) the PPQ fails to cover the construct of personally experienced stress, (3) it is unknown how publication pressure relates to general work pressure in academics, and (4) the PPQ is particularly focused on (bio) medical research(ers).

Firstly, although intended to assess publication pressure, the PPQ items mostly ask about the researchers’ attitude regarding current publication culture. The fact that a researcher perceives the current publication culture as negative does not necessarily result in severe pressure. Since the majority of the PPQ items do not reflect the core question (does this researcher experience publication pressure and if so, how much?), then this ambiguity in interpretation of the PPQ sum score threatens the content validity of the PPQ.

Secondly, in the PPQ’s validation study, the relationship between the PPQ and the Maslach Burnout Inventory (MBI) [11] was investigated. The PPQ correlated only moderately with relevant MBI subscale scores (*r* = 0.34 with emotional exhaustion and *r* = 0.31 with depersonalisation). Yet, a meta-analysis by Lee and Ashfort found all relevant job stressors to correlate above 0.5 with emotional exhaustion [12]. If burnout is a feasible outcome of publication pressure [8], one would expect correlations to be higher. Gillespie et al. found that among two thirds of the academics they consulted in their study described psychological problems resulting from stress, with burnout featuring prominently [13]. If the PPQ scores are not consistent with initial ideas about its relationship with burnout, then this leaves doubts about the convergent construct validity of the PPQ.

Thirdly, publication pressure could be viewed as one aspect of work pressure in academics, which raises the question how (the measurement of) publication pressure relates to (more general measurements of) work pressure, which is one of the most important research areas in work psychology [14]. There are interesting parallels with work pressure and publication pressure. Both work pressure and publication pressure may lead to burnout-like symptoms and both may encourage one to think about potential misbehaviour [15–18]. Yet, there is little mentioning in research integrity literature of some of the prominent work stress models from psychology [19]. As the PPQ was initially designed to study the effect of publication pressure on researchers’ tendency to misbehave, work stress models may be a helpful extension for studying the effect of publication pressure on researchers and their integrity [8].

In addition, how does publication pressure relate to other well-known causes of stress and burnout, such as work-home interference and job insecurity (the fear of losing one’s job)? Both work-home interference and job insecurity seem highly relevant in academic researchers [13, 20, 21]. Since the PPQ does not mention any of these (arguably relevant) constructs, that leaves its divergent construct validity to be desired.

Lastly, the PPQ was constructed and tested in professors working in biomedicine. Arguably, biomedical professors constitute only a small subset of the total population of academic researchers that may experience publication pressure. Some PPQ items explicitly mention the medical field (e.g. ‘My scientific publications contribute to better (future) medical care’). Since the current phrasing is tailored to biomedical researchers, it is hard to assess the generalisability of the results in other academic disciplines, lowering the external validity of the PPQ.

These validity issues formed the motivation for a revision of the PPQ. Below, we first present the methods used to revise the PPQr and to construct new items. Second, we assess the factorial structure and examine the reliability and (internal and external) validity of the new PPQr subscales, by calculating Cronbach’s alphas and correlations between the PPQr subscales and relevant work pressure and burnout constructs. Finally, we administer the PPQr in a larger, independent sample of researchers to check its reliability.

### Study’s aim

This study’s aim is threefold. First, to revise the PPQ and address the abovementioned concerns that should lead to the design of the revised version of the PPQ (the PPQr, see the ‘Instrument construction’ section). Second, to study the PPQr in relation to work pressure and burnout (see the ‘Pilot study’ section). Lastly, we want to redistribute the PPQr in an independent sample and test the presupposed structure and reliability in a more diverse group of academics (see the ‘Reliability study’ section).

## Methods

### Instrument construction

In work psychology literature, stress and the consequences of stress at work are one of the most frequently studied topics [14]. One prominent conception of how stress is moderated stems from the Job Demands-Control model (JDC) [22], the Effort-Reward Imbalance model (ERI) [23], and later the Job Demands-Resources model of burnout (JD-R) [24]. These models propose that the balance between positive and negative work characteristics is important for various work outcomes. As a result, stress is seen as an interplay between (high) job demands and (low) job resources [24].

Within the JD-R model, demands refer to physical, social, or organisational aspects of the job that require sustained effort, such as work pressure, ambiguity about an employee’s role, or stressful events in general. Resources on the other hand refer to aspects of the job that are helpful in achieving work goals, stimulate development, and reduce the costs of job demands. Examples of resources are social support from family or colleagues, possibilities for career development, or autonomy. Job demands and resources interact; job resources can buffer the impact of job demands in predicting employee health and motivation. In a nutshell, when demands exceed resources, someone is likely to perceive stress or even burnout symptoms [24].

There are warning signs that burnout is a growing problem in academia [11]. A Flemish study found 50% of PhD students to face psychological distress which caused them to be more at risk for developing burnout compared to the general higher educated population [6]. Moreover, a UK study demonstrated that 15% of academics experience such profound levels of stress that they needed medical advice [25] and roughly one in five Dutch medical professors met official burnout criteria [7].

Based on the literature on stress and burnout, we tried to determine what content should be included in an instrument that can measure publication pressure. The PPQ mostly enquired attitudes towards the current publication culture, but items about perceived publication stress or publication resources were missing. As a consistent definition of work stress in academia is lacking [26], we identified possible job demands for academic researchers inspired by the Job Content Questionnaire [27], i.e. (lack of) social support, (lack of) autonomy, authority, psychological demands, and skills, respectively. All these constructs have been extensively studied in relation to stress and burnout [28].

To assure content validity, we used the facet method to formulate new items [29]. The facet method strengthens content validity by structuring the analysis of the concept one wishes to study [30]. We used the facet method to assure that we did not miss any relevant aspect of publication pressure. Relevant work stress characteristics, applied to publishing, appear in the left column of Additional file 1. The top row of Additional file 1 specifies two types of experiences: first, whether respondents experience stress, and second, whether respondents experience lack of resources based on the Perceived Stress Scale [31]. In total, we formulated 37 items, with the aim of ending with a shorter and more user-friendly questionnaire. We kept the response options for the PPQr the same as the original PPQ: items are scored on a 5-point Likert scale (1 = ‘totally disagree’, 5 = ‘totally agree’).

To check whether our drafted items were understandable and generalisable, we asked PhD candidates and assistant professors from biomedical and behavioural sciences to test and inspect the items for comprehensiveness (*n* = 9). This resulted in minor modifications in wording to improve clarity and correct interpretation.

### Pilot study

#### Materials

In addition to the PPQr items and questions about demographics, we included the complete Maslach Burnout Inventory-General Survey (MBI) [11], subscales of the Work Design Questionnaire (WDQ) [32] and the Job Insecurity Scale (JIS) [33], and items about negative work-home interference taken from the Survey Work-home Interaction—NijmeGen (SWING) [34].

The MBI was included to measure burnout and stress and to examine the PPQr’s convergent construct validity. Being the most used instrument to measure burnout, the MBI consists of 22 items spread over three subscales: emotional exhaustion (9 items, *α* = 0.90), depersonalisation (5 items, *α* = 0.80), and personal accomplishment (8 items, *α* = 0.73). Emotional exhaustion is the feeling of depletion of energy during work and a negative attitude towards work-related activities. Depersonalisation is the alienation from work, where someone’s interest in work or colleagues is completely lost. Personal accomplishment is a positive subscale; it regards the feelings of content and a sense of being capable to do the work. Responses are scored on a Likert scale from 1 ('never') to 5 ('every day') [11].

We chose the WDQ to measure the PPQr’s divergent construct validity in relation to work pressure [32]. Not all subscales of the WDQ are relevant to working in academia (contextual characteristics such as physical demands are arguably not relevant to all academics), so we chose a selection of the WDQ items from categories task characteristics (12 items), knowledge characteristics (12 items), and social characteristics (9 items). From task characteristics, we took subscales work scheduling autonomy (*α* = 0.85), decision-making autonomy (*α* = 0.85), and work methods autonomy (*α* = 0.88) and feedback from work (*α* = 0.86). From knowledge characteristics, we took information processing (*α* = 0.87), problem solving (*α* = 0.84), and specialisation (*α* = 0.84). Finally, we took two subscales from social characteristics, namely social support (*α* = 0.82) and feedback from others (*α* = 0.88). All items are scored on a 5-point scale from ‘strongly disagree’ to ‘strongly agree’.

Two other constructs commonly referred to as causes of stress and burnout in academics are job insecurity and negative work-home interference. Job insecurity is the subjective feeling that you can lose your job at any point [33, 35]. The Job Insecurity Scale measures personally experienced job insecurity. The questionnaire consists of one subscale that measures the perceived threat of losing one’s job and the worries that accompany this threat (4 items). The reliability of this scale is good (*α* = 0.82). Answers are scored on a 5-point Likert scale with 1 being ‘strongly disagree’ and 5 ‘strongly agree’.

Negative work-home interference regards the hindrance that people experience at home as a result of their work. Typical examples entail over-working, staying at work for long hours during the week, or having to always work on the weekends. We used the subscale work-home interference (9 items) of the Survey Work-home Interaction—NijmeGen (SWING) [34]. Reliability of the negative work-home interference is good (*α* = 0.85). Answers indicate how often participants experience certain situations on a 4-point scale from ‘practically never’ (0) to ‘practically always’ (3).

#### Procedure

We distributed the survey through our own network and social media. The survey was available via an online link through Qualtrics. The questionnaire included (parts of) existing instruments (complete MBI, relevant parts of the WDQ, JIS, and SWING) as well as the PPQr items and demographics. All items were in English. After reading about the purpose and procedure of the study, participants had to give informed consent before continuing to the actual questions. The questionnaire took approximately 15 min to complete.

#### Participants

All researchers (including PhD students) currently employed at an academic institution were eligible to participate. Two hundred five researchers started the questionnaire, 129 respondents provided enough useful answers to include them for analyses, and 66% of those was female. The majority of the respondents worked in biomedicine (52%), besides 43% worked in social science and 5% had a background in natural sciences or humanities. Thirty-eight percent of the participants were PhD student, 24% was currently employed as a postdoctoral researcher, 20% as assistant professor and 19% associate or full professor. The average age was 37.

## Results

In order to assess which items best measured publication pressure, we carried out a principal component analysis (PCA). The first three components were identified as ‘stress’, ‘attitude’, and ‘resources’. We selected 3 × 6 = 18 items with high loadings in the three-component solution, but not necessarily the items with the highest loadings because we tried to cover as many aspects as relevant to experiencing lack of resources when working on publications or experiencing stress when working on publishing. See Additional file 2 for the pattern matrix with the three components. See Table 1 for an overview of selected items per subscale.

**Table 1:** PPQr subscales’ items with alphas, means, standard deviations, and item-rest correlations

Cronbach’s alpha	Mean*	SD*	r*	Items
Stress (*α* = 0.80)	3.22	0.80		Stress subscale
	2.98	1.22	0.55	I experience stress at the thought of my colleagues’ assessment of my publications output.
	3.88	1.09	0.44	I feel forced to spend time on my publications outside office hours.
	3.52	1.10	0.43	I cannot find sufficient time to work on my publications.
	2.79	1.12	0.60	I have no peace of mind when working on my publications.
	2.87	1.01	0.50	I can combine working on my publications with my other tasks.
	3.27	1.12	0.57	At home, I do not feel stressed about my publications.
Attitude (*α* = 0.78)	3.59	0.68		Attitude subscale
	3.39	1.10	0.46	The current publication climate puts pressure on relationships with fellow-researchers.
	3.84	0.94	0.47	I suspect that publication pressure leads some colleagues (whether intentionally or not) to cut corners.
	3.41	1.08	0.46	In my opinion the pressure to publish scientific articles has become too high.
	3.93	0.93	0.50	My colleagues judge me mainly on the basis of my publications.
	2.99	0.98	0.40	Colleagues maintain their administrative and teaching skills well, despite publication pressure.
	4.01	0.93	0.47	Publication pressure harms science.
Resources (*α* = 0.75)	2.21	0.63		Resources subscale
	2.09	0.82	0.45	When working on a publication, I feel supported by my co-authors.
	1.84	0.78	0.42	When I encounter difficulties when working on a publication, I can discuss these with my colleagues.
	2.26	1.04	0.39	I have freedom to decide about the topics of my publications.
	2.30	1.00	0.37	When working on a publication, many decisions about the content of the paper are outside my control.
	2.46	1.05	0.50	I cannot cope with all aspects of publishing my papers.
	2.31	0.90	0.46	I feel confident in the interaction with co-authors, reviewers and editors.

*These means, standard deviations, and item-rest correlations are taken from the reliability sample

Item-rest correlations (aka corrected item-total correlations) for stress were between 0.44 and 0.66. Attitude item-rest correlations ranged from 0.38 to 0.60. Finally, resources’ items correlated between 0.30 and 0.54 with their subscale.

We calculated the reliability of the subscales. Cronbach’s alphas were 0.83 for stress, 0.80 for attitude, and 0.76 for resources, which is considered acceptable [36].

Items were recoded in such a way that higher subscale scores indicate more publication pressure. A respondent that scores low on all subscales experiences little stress from publishing, has a positive attitude about publishing, and has sufficient resources.

We checked the validity of the PPQr by inspecting the correlations with the MBI, the WDQ, the JHI, and the WHI. Stress correlated 0.62 with MBI’s emotional exhaustion and 0.46 with the total MBI. Work-home interference and stress were also highly correlated (*r* = 0.69). Resources correlated between − 0.41 and − 0.50 with relevant included WDQ subscales and moderately with job insecurity (*r* = 0.33). For a full overview of subscale correlations, see Table 2. For PPQr items’ correlations with PPQr subscales, see Additional file 3.

**Table 2:** Correlations between included constructs

Constructs and subscales	PPQr stress	PPQr attitude	PPQr resources
MBI total	0.46	0.32	0.19
MBI—emotional exhaustion	0.62	0.42	0.34
MBI—depersonalisation	0.33	0.37	0.38
MBI—personal accomplishment	− 0.22	− 0.23	− 0.40
WDQ—task	− 0.37	− 0.22	− 0.47
WDQ—knowledge	0.25	0.06	− 0.05
WDQ—feedback	− 0.30	− 0.48	− 0.41
WDQ—social	− 0.30	− 0.39	− 0.50
Job insecurity	0.17	0.17	0.33
Work-home interference	0.69	0.41	0.31
PPQ—stress	1	0.48	0.43
PPQ—attitude	0.48	1	0.36
PPQ—resources	0.43	0.36	1

Note: sample size is 129

To assess the added value of publication pressure as an indicator of burnout, we conducted hierarchical regression analyses with emotional exhaustion (the most prototypical burnout indicator from the MBI) as the outcome variable. Various predictor selection procedures yielded the same result. We found emotional exhaustion to be best predicted by work-home interference, followed by social support and publication stress (*r*^*2*^ = 0.59). This indicates that publication stress is a relevant indicator of burnout, even when considering the influence of other burnout predictors such as work-home interference and (lack of) social support. See Additional file 4 for the prediction model(s).

We conclude that the PPQr is sufficiently reliable (all Cronbach’s alphas > 0.7; [36]) and construct validity is also good, as evidenced by its strong correlations with the relevant MBI and WDQ subscales. As publication stress is a significant predictor of burnout, this indicates good predictive validity.

Still, these are preliminary conclusions, as we used a single sample for both item selection and reliability and validity analysis. In order to check whether the proposed structure and reliability would hold, we administered the PPQr in a large and independent sample, as part of a study investigating the academic research climate [37], see www.amsterdamresearchclimate.nl.

### Reliability study

#### Materials

Besides the PPQr (18 items) and demographics, the survey contained the Survey of Organisational Research Climate (SOuRCe) [38] and a list of 60 major and minor misbehaviours [39]. In this paper, we only report about the structure and reliability of the PPQr.

#### Procedure

We obtained ethical approval from the Scientific and Ethical Review Board of the Faculty of Behavioural and Movement Sciences from the VU University Amsterdam. A data sharing agreement with participating institutions University of Amsterdam, Amsterdam Medical Centre, and VU University Medical Centre secured the e-mail addresses of all academic researchers. We designed and distributed the survey using Qualtrics.

First, we sent an information letter to explain the purpose of the study. The survey questionnaire was sent out over e-mail and started when participants provided informed consent. The complete questionnaire took about 15 to 20 min to complete. We sent three reminders, each 10 days apart.

#### Participants

All academic researchers, employed at an academic institute in Amsterdam between May 1 and July 18, 2017, were eligible to participate. This again included PhD students. To be eligible for inclusion, a respondent had to be involved in research for at least 1 day per week. One thousand sixty-three academic researchers completed the PPQr (59% women). Fifty-six percent worked in biomedicine, 23% was from the social sciences, and the remaining 21% from the natural sciences and humanities. Forty-nine percent was PhD candidate, 30% postdoc or assistant professor, and the remaining 21% associate or full professor.

#### Results

A total of 7549 academic researchers were invited to participate in the study, of which 1063 completed the full PPQr (14%). First, we wanted to assess the internal structure of the PPQr by means of item-correlations and principal component analysis. Second, we aim to assess whether the PPQr is reliable by computing Cronbach’s alpha coefficients for each of the PPQr’s three subscales.

To assess the internal structure of the PPQr, we conducted a principal component analysis. The three-component solution explained 50% of the variance, and the scree plot also indicates a three-component solution. The pattern matrix showed that each item has the highest loadings on its own component (see Additional file 5). In addition, we conducted confirmatory factor analyses (CFA) which showed that a three-factor model fitted the data of the full sample satisfactorily and that the same three-factor model also fitted the data of each of the subgroups of men and women, four disciplines, and five academic ranks.

Corrected item-subscale correlations for attitude ranged between 0.40 and 0.50. For stress, this was slightly higher: 0.43 and 0.60. For resources, item-subscale correlations were between 0.37 and 0.50. Cronbach’s alphas were 0.80, 0.78, and 0.75 for stress, attitude, and resources, respectively. We also calculated Cronbach’s alphas for subgroups of men and women, four disciplines, and five academic ranks, but subgroup results did not substantially deviate from the full sample results. Correlations between the subscales were 0.46 between stress and attitude, 0.44 between stress and resources, and 0.39 between attitude and resources.

We conclude that the PPQr is a robust instrument to measure publication pressure in academic researchers.

## Discussion

We aimed to improve the PPQ in order to accommodate concerns about its validity and created a revised version of the PPQ (PPQr). This new instrument (18 items) consists of three subscales: publication stress (6 items), publication attitude (6 items), and publication resources (6 items). After validating the PPQr in a convenience sample, we tested the reliability of the PPQr in an independent sample.

We conclude that the PPQr is a valid instrument; correlations with both MBI subscales and relevant WDQ subscales are substantial and in the expected direction (all relevant *r* > 0.4). Each of these subscales is reliable (all Cronbach’s alpha > 0.7). The PPQr can be used to study publication pressure among academic researchers from all disciplinary fields and academic ranks.

This enables us to investigate the relation between publication pressure and work stressors. The PPQr is strongly related to work pressure (correlations resources and relevant WDQ subscales between 0.41 and 0.50), yet publication pressure seems at least in some ways to differ from ‘classical’ work pressure as it was only marginally related to the knowledge characteristics subscale of the WDQ (see Table 2). Furthermore, subscale resources underscores the relation between publication pressure and job insecurity, a researcher with less resources is more likely to experience job insecurity (or conversely, a researcher with low job insecurity is more likely to perceive resources). Stress is strongly associated with work-home interference, a researcher who experiences more work-home interference is more likely to experience publication stress (and vice versa).

Hierarchical regression analyses indicated that publication pressure was strongly related to burnout; hence, a researcher who perceives higher publication pressure may be *more likely* to develop burnout symptoms. With the PPQr, this relation becomes even more apparent than with the PPQ since its correlations with the MBI subscale emotional exhaustion are stronger than the PPQ (*r* = 0.34 for the original PPQ and *r* = 0.62 for the PPQr).

However, work-home interference was more strongly related to burnout in our sample (*r* = 0.73 and *r*^*2*^ = 0.53, *p* < 0.001, see Additional file 4). This can be expected as work-home interference is known to be directly associated with burnout [40]. Nevertheless, adding publication stress to the hierarchical regression model significantly increased the explained variance, emphasising its importance besides other burnout markers.

### Alternative explanations

It could be that publication pressure is determined by factors currently not included in the PPQr; two particularly important ones being the acquisition pressure and pressure from teaching duties. Along these lines, role conflict (the reasoning here is that since people have a limited amount of time and multiple tasks or responsibilities, when one task requires major attention, the other tasks suffer since there is simply no more time or attention left) is known to be a predictor of work stress. In this situation, the internal role conflict would regard academics to both be good researchers and good teachers. We did not measure role conflict in our study, yet it seems plausible that role conflict would lead to burnout and not so much publication pressure per se. We encourage future research into the relationship between evaluation criteria and role conflict in relation to publication pressure.

Another alternative explanation would be that publication pressure is mostly dependent on evaluation criteria as set by the institution of employment. To put it simple, a postdoc that needs to publish 10 papers a year will feel more publication pressure than a postdoc who is evaluated based on just 3 papers a year. A complicating factor herein is that it is nearly impossible to access individual evaluation criteria. Nevertheless, it would be fruitful in future research to study PPQr scores in relation to the amounts of papers a researcher is expected to publish within a specific timeframe to attribute and interpret the score of the three subscales for an individual researcher to develop cut-off scores.

Finally, it could be that researchers with burnout symptoms experience more pressure and annoyance from the current publication system because of their symptoms, so in this conceptualisation, burnout precedes publication pressure. Alternatively, since there is an abundance of research indicating that high job demands increase the risk of developing burnout symptoms, it could be that both publication stress and burnout are the results of excessive job demands or a related variable. We cannot exclude these possibilities based on our data and would encourage longitudinal investigation into this matter so to confirm that publication pressure precedes burnout or vice versa.

### Strengths

This study moves away from operationalizing publication pressure as an attitude or opinion of its severity to extended operationalisation towards personally experienced pressure. Individual experience, not opinion, is one of the strongest driving forces of behaviour [41]; someone can think that publication pressure pushes researchers beyond limits of responsible research, yet if that person herself does not lay awake at night because of her *H*-index, there seems little reason to suspect burnout is looming.

Secondly, the large sample (> 1000 academic researchers) may increase the reliability of the results. Our sample consists of academic researchers from all academic ranks and disciplinary field, which should indicate better generalisability.

### Limitations

Most evident is the use of a convenience sample for the pilot study. We recruited respondents by means of our personal network and social media. This can result in a selective pilot sample. Still, we found similar results in the reliability study, with an independent sample.

Secondly, the response rate (14%) for our reliability study is low. This could increase the chance of a response bias, which occurs when responders differ critically from non-responders. Statistics on female academics in The Netherlands indicate women make up 39% of the academic workforce, whereas 59% of our participants identified as female. Similarly, national statistics indicate that 30% of academic researchers are currently enrolled as a (non-biomedical) PhD candidate compared to 41% in our sample. Yet, this would only indicate response bias if the PPQr items were understood differently depending on one’s subgroup. Since the CFA model fit did not differ significantly between different subgroups, we conclude that this should not affect the validity of the PPQr results we present here.

Another limitation is that the current PPQr cannot be expressed in one total score as was the case with its predecessor. We intended to make a total score to ease interpretation. However, upon reflection, it is unclear what that total score would express and hence we decided against it.

## Conclusion

The PPQr is a valid and reliable measurement instrument. It covers the complex construct of publication pressure better than its predecessor and can measure publication pressure among researchers from all disciplinary fields. PPQr scores are strongly related to emotional exhaustion scores. The PPQr could also be beneficial for policy makers and research institutions to assess the degree of publication pressure in their institute. To sustain responsible research, institutions should invest in resources to combat the high demands, such as fostering an open atmosphere where difficulties can be discussed and where researchers have some freedom in deciding what to study.

## Additional files

Additional file 1: **Table S1.** Newly developed PPQr items using the facet method. (DOCX 14 kb)

Additional file 2: **Table S2.** Pattern matrix with 3 components. (DOCX 18 kb)

Additional file 3: **Table S3.** Correlations PPQr items with PPQr subscales. (DOCX 15 kb)

Additional file 4: **Table S4.** Regression model to predict emotional exhaustion (MBI). (DOCX 14 kb)

Additional file 5: **Table S5.** Pattern matrix with PPQ items and 3 components taken from the reliability sample. (DOCX 16 kb)

## Abbreviations

CFA: Confirmatory factor analysis
ERI: Effort-Reward Imbalance model
JDC: Job Demands-Control model
JD-R: Job Demands-Resources model
JIS: Job Insecurity Scale
MBI: Maslach Burnout Inventory
PCA: Principal component analysis
PPQ: Publication Pressure Questionnaire
PPQr: Revised Publication Pressure Questionnaire
SWING: Survey Work-home Interaction—NijmeGen
WDQ: Work Design Questionnaire
