# Supplementary material for: Personally perceived publication pressure: revising the Publication Pressure Questionnaire (PPQ) by using work stress models
Source: Res Integr Peer Rev. 2019 Apr 9;4:7. doi: 10.1186/s41073-019-0066-6 (PMC6454769; doi:10.1186/s41073-019-0066-6)
Supplement: Supplementary file 1 — Table S1. Newly developed PPQr items using the facet method. (DOCX 14 kb) [file 41073_2019_66_MOESM1_ESM.docx]

**Table 1**. Newly developed PPQr items using the facet method.

|  | **Experiencing lack of resources** | **Experiencing stress** |
| --- | --- | --- |
| **Social Support** | - When working on a publication, I feel supported by my co-authors.  - My immediate supervisor understands the problems I encounter when I work on my publications.  - When I encounter difficulties when working on a publication, I can discuss these with my colleagues. | - I get irritated when collaborating with co-authors.  - I get nervous when I discuss my publications with colleagues. |
| **Autonomy** | - I can decide how much time I spend on my publications.  - I have the possibility to work on my publications at home if I would like to.  - I feel forced to spend time on my publications outside office hours. | - I cannot find sufficient time to work on my publications.  - I have no peace of mind when working on my publications. |
| **Authority** | - I have freedom to decide about the topics of my publications.  - When working on a publication, many decisions about the content of the paper are outside my control.  - When revising my publications, I can address the comments of the reviewers and editors in the way I would like. | - I am not able to control all aspects of publishing papers. |
| **Psychological Demands** | - I have difficulty finishing my publications in time.  - I feel I should publish more than I do now.  - I can combine working on my publications with my other tasks. | - At home, I do not feel stressed about my publications.  - I am unable to juggle the work on my publications with my other priorities. |
| **Skills** | - I feel sufficiently capable to write an academic paper. | - I cannot cope with all aspects of publishing my papers.  - I feel confident in the interaction with co-authors, reviewers and editors.  - I cannot overcome all difficulties of publishing my papers. |
