# Supplementary material for: Personally perceived publication pressure: revising the Publication Pressure Questionnaire (PPQ) by using work stress models
Source: Res Integr Peer Rev. 2019 Apr 9;4:7. doi: 10.1186/s41073-019-0066-6 (PMC6454769; doi:10.1186/s41073-019-0066-6)
Supplement: Supplementary file 2 — Table S2. Pattern matrix with 3 components. (DOCX 18 kb) [file 41073_2019_66_MOESM2_ESM.docx]

**Table 2.** Pattern Matrix with 3 components.

| **PPQr items** | **Components*** | | |
| --- | --- | --- | --- |
|  | **1 ‘Stress’** | **2 ‘Attitude’** | **3 ‘Resources’** |
| 1. Without publication pressure, my scientific output would be of higher quality. | .134 | .501 | .201 |
| 1. My publications contribute to scientific progress. | -.032 | .221 | .304 |
| 1. **I experience stress at the thought of my colleagues' assessment of my publications output.** | **.537** | .233 | .050 |
| 1. I experience the publication criteria formulated by my employer as a positive motivating factor. | .175 | .671 | -.070 |
| 1. **The current publication climate puts pressure on relationships with fellow-researchers.** | .050 | **.541** | -.010 |
| 1. **I suspect that publication pressure leads some colleagues (whether intentionally or not) to cut corners.** | -.134 | **.722** | .082 |
| 1. Publication pressure in university institutions increases the validity of scientific literature. | -.119 | .783 | -.201 |
| 1. Publication pressure leads to questionable research practices, which lead to serious doubts about the validity of research results. | -.155 | .763 | .043 |
| 1. **In my opinion the pressure to publish scientific articles has become too high.** | .173 | **.708** | .000 |
| 1. The competitive scientific climate pressures me to publish more. | .249 | .015 | .075 |
| 1. **My colleagues judge me mainly on the basis of my publications.** | .330 | **.320** | .043 |
| 1. **Colleagues maintain their administrative and teaching skills well, despite publication pressure.** | .174 | **.421** | -.033 |
| 1. Because of publication pressure, I cannot confide innovative research proposals to my colleagues. | .292 | .402 | .091 |
| 1. **Publication pressure harms science.** | .089 | **.829** | -.059 |
| 1. **When working on a publication, I feel supported by my co-authors.** | .052 | .208 | **.462** |
| 1. My immediate supervisor understands the problems I encounter when I work on my publications. | .152 | .233 | .047 |
| 1. **When I encounter difficulties when working on a publication, I can discuss these with my colleagues.** | .105 | .143 | **.386** |
| 1. I get irritated when collaborating with coauthors. | .065 | .477 | .198 |
| 1. I get nervous when I discuss my publications with colleagues. | .361 | .101 | .338 |
| 1. I can decide how much time I spend on my publications. | .349 | .303 | .177 |
| 1. I have the possibility to work on my publications at home if I would like to. | .200 | -.082 | .415 |
| 1. **I feel forced to spend time on my publications outside office hours.** | **.640** | .063 | .027 |
| 1. **I cannot find sufficient time to work on my publications.** | **.739** | .237 | -.154 |
| 1. **I have no peace of mind when working on my publications.** | **.589** | .175 | .224 |
| 1. **I have freedom to decide about the topics of my publications.** | -.132 | -.110 | **.774** |
| 1. **When working on a publication, many decisions about the content of the paper are outside my control.** | -.050 | .048 | **.755** |
| 1. When revising my publications, I can address the comments of the reviewers and editors in the way I would like. | -.086 | -.064 | .683 |
| 1. I am not able to control all aspects of publishing papers. | -.240 | .223 | .281 |
| 1. I have difficulty finishing my publications in time. | .635 | .021 | .062 |
| 1. I feel I should publish more than I do now. | .733 | -.219 | .063 |
| 1. **I can combine working on my publications with my other tasks.** | **.784** | -.009 | -.118 |
| 1. **At home, I do not feel stressed about my publications.** | **.540** | .039 | .239 |
| 1. I am unable to juggle the work on my publications with my other priorities. | .644 | .047 | -.037 |
| 1. I feel sufficiently capable to write an academic paper. | .042 | -.161 | .600 |
| 1. **I cannot cope with all aspects of publishing my papers.** | .282 | -.032 | **.543** |
| 1. **I feel confident in the interaction with co-authors, reviewers, and editors.** | .123 | .121 | **.582** |
| 1. I cannot overcome all difficulties of publishing my papers. | .141 | .036 | .505 |

*Rotation method: Oblimin with Kaiser Normalisation. Selected items are printed in **bold**.
