# Supplementary material for: Personally perceived publication pressure: revising the Publication Pressure Questionnaire (PPQ) by using work stress models
Source: Res Integr Peer Rev. 2019 Apr 9;4:7. doi: 10.1186/s41073-019-0066-6 (PMC6454769; doi:10.1186/s41073-019-0066-6)
Supplement: Supplementary file 3 — Table S3. Correlations PPQr items with PPQr subscales. (DOCX 15 kb) [file 41073_2019_66_MOESM3_ESM.docx]

**Table 3**. Correlations PPQr items with PPQr subscales.

| **PPQ #** | **PPQ item written out** | **Stress** | **Attitude** | **Resources** |
| --- | --- | --- | --- | --- |
| 1 | I experience stress at the thought of my colleagues' assessment of my publications output. | **.713**** | .433** | .271** |
| 2 | I feel forced to spend time on my publications outside office hours. | **.715**** | .279** | .336** |
| 3 | I cannot find sufficient time to work on my publications. | **.783**** | .437** | .222 |
| 4 | I have no peace of mind  when working on my publications. | **.786**** | .407** | .471** |
| 5 | I can combine working on my publications with my other tasks. | **.719**** | .239* | .190 |
| 6 | At home, I do not feel stressed about my publications. | **.717**** | .292** | .411** |
| 7 | The current publication climate puts pressure on relationships with fellow-researchers. | .303** | **.621**** | .239** |
| 8 | I suspect that publication pressure leads some colleagues (whether intentionally or not) to cut corners. | .261** | **.739**** | .265** |
| 9 | Publication pressure leads to questionable research practices, which lead to serious doubts about the validity of research results | .261** | **.718**** | .224* |
| 10 | In my opinion the pressure to publish scientific articles has become too high. | .483** | **.758**** | .282** |
| 11 | Colleagues maintain their administrative and teaching skills well, despite publication pressure. | .273** | .**579**** | .234** |
| 12 | Publication pressure harms science. | .445** | **.838**** | .269** |
| 13 | When working on a publication, I feel supported by my co-authors. | .300** | .274** | **.685**** |
| 14 | When I encounter difficulties when working on a publication, I can discuss these with my colleagues. | .276** | .256** | **.609**** |
| 15 | I have freedom to decide about the topics of my publications. | .125 | .097 | **.706**** |
| 16 | When working on a publication, many decisions about the content of the paper are outside my control. | .293** | .246* | **.776**** |
| 17 | I cannot cope with all aspects of publishing my papers. | .378** | .222 | **.602**** |
| 18 | I feel confident in the interaction with co-authors, reviewers and editors. | .402** | .379** | **.665**** |

* indicates *p* < .05, ** indicates *p* <.001.
