# Supplementary material for: Personally perceived publication pressure: revising the Publication Pressure Questionnaire (PPQ) by using work stress models
Source: Res Integr Peer Rev. 2019 Apr 9;4:7. doi: 10.1186/s41073-019-0066-6 (PMC6454769; doi:10.1186/s41073-019-0066-6)
Supplement: Supplementary file 4 — Table S4. Regression model to predict emotional exhaustion (MBI). (DOCX 14 kb) [file 41073_2019_66_MOESM4_ESM.docx]

**Table 4.** Regression model to predict Emotional Exhaustion (outcome variable) as measured by the MBI.

| **Model** | ***R*^2^** | **Variables included** | ***β coefficient*** | ***Standard deviation*** | ***p*-value** |
| --- | --- | --- | --- | --- | --- |
| 1 | .53 | Constant | .169 | .257 | .511 |
|  |  | Work-home Interference | 1.285 | .108 | .000 |
| 2 | .58 | Constant | 2.283 | .598 | .000 |
|  |  | Work-home Interference | 1.177 | .106 | .000 |
|  |  | Social Support | -.472 | .122 | .000 |
| 3 | .598* | Constant | 1.790 | .627 | .005 |
|  |  | Work-home Interference^#^ | .962 | .141 | .000 |
|  |  | Social Support | -.427 | .122 | .001 |
|  |  | Publication Stress | .244 | .107 | .024 |

* Increase in *R*^2^ was significant (*p* = .017). No other variable significantly improved the explanatory value.

^#^ Note: we also investigated interaction effects of gender but these were not significant.
