# Supplementary material for: Personally perceived publication pressure: revising the Publication Pressure Questionnaire (PPQ) by using work stress models
Source: Res Integr Peer Rev. 2019 Apr 9;4:7. doi: 10.1186/s41073-019-0066-6 (PMC6454769; doi:10.1186/s41073-019-0066-6)
Supplement: Supplementary file 5 — Table S5. Pattern matrix with PPQ items and 3 components taken from the reliability sample. (DOCX 16 kb) [file 41073_2019_66_MOESM5_ESM.docx]

**Table 5.** Pattern matrix with PPQ items and 3 components taken from the reliability sample.

| **PPQ item*** | **Component 1** ‘Stress’** | **Component 2** ‘Attitude’** | **Component 3** ‘Resources’** |
| --- | --- | --- | --- |
| Publication Stress 1 | .466 | .123 | -.252 |
| Publication Stress 2 | .711 | .123 | .164 |
| Publication Stress 3 | .831 | -.034 | .135 |
| Publication Stress 4 | .595 | .015 | -.295 |
| Publication Stress 5 | .752 | -.045 | -.051 |
| Publication Stress 6 | .587 | .102 | -.169 |
| Publication Attitude 1 | .141 | .600 | .030 |
| Publication Attitude 2 | -.025 | .784 | .037 |
| Publication Attitude 3 | -.142 | .816 | -.055 |
| Publication Attitude 4 | .115 | .745 | .097 |
| Publication Attitude 5 | .191 | .312 | -.131 |
| Publication Attitude 6 | -.073 | .716 | -.085 |
| Publication Resources 1 | -.058 | -.027 | .659 |
| Publication Resources 2 | -.164 | .056 | .582 |
| Publication Resources 3 | .101 | -.003 | .743 |
| Publication Resources 4 | .156 | -.020 | .751 |
| Publication Resources 5 | -.281 | .007 | .510 |
| Publication Resources 6 | .001 | -.134 | .617 |

* For written items, see **Table 2** main text. ** Rotation Method: Oblimin with Kaiser Normalization.

A full report of the CFA and subsequent measurement invariance analyses would be beyond the scope of the paper. Still, we do want to inform the interested reader about the results. A simple three factor model did not fit satisfactorily (CHISQ(132) = 1126.9, RMSEA = 0.086) because a few inter-item correlations were somewhat higher than expected under the simple model. After accounting for these violations of local independence, which are inconsequential for PCA’s and reliability, the fit was satisfactory (CHISQ(128) = 565.8, RMSEA = 0.057). This model also yielded satisfactory fit to each of the subgroups (men and women, four disciplines, and five academic ranks).

Moreover, hypotheses of invariance of factor loadings and intercepts were not rejected according to RMSEA and ECVI differences, except for the intercepts of three stress items that indicated that, if stress levels would be equal, these items were easier to agree with for PhD students than for other subgroups.
